# First person – Takashi Owaki

**DOI:** 10.1242/dmm.049734

**Published:** 2022-07-26

## Abstract

First Person is a series of interviews with the first authors of a selection of papers published in Disease Models & Mechanisms, helping early-career researchers promote themselves alongside their papers. Takashi Owaki is first author on ‘ Involvement of the liver-gut peripheral neural axis in nonalcoholic fatty liver disease pathologies via hepatic HTR2A’, published in DMM. Takashi is an MD and PhD student in the lab of Kenya Kamimura and Professor Shuji Terai at Niigata University, Niigata, Japan, investigating the effect of inter-organ communication on liver diseases via the autonomic nervous system.


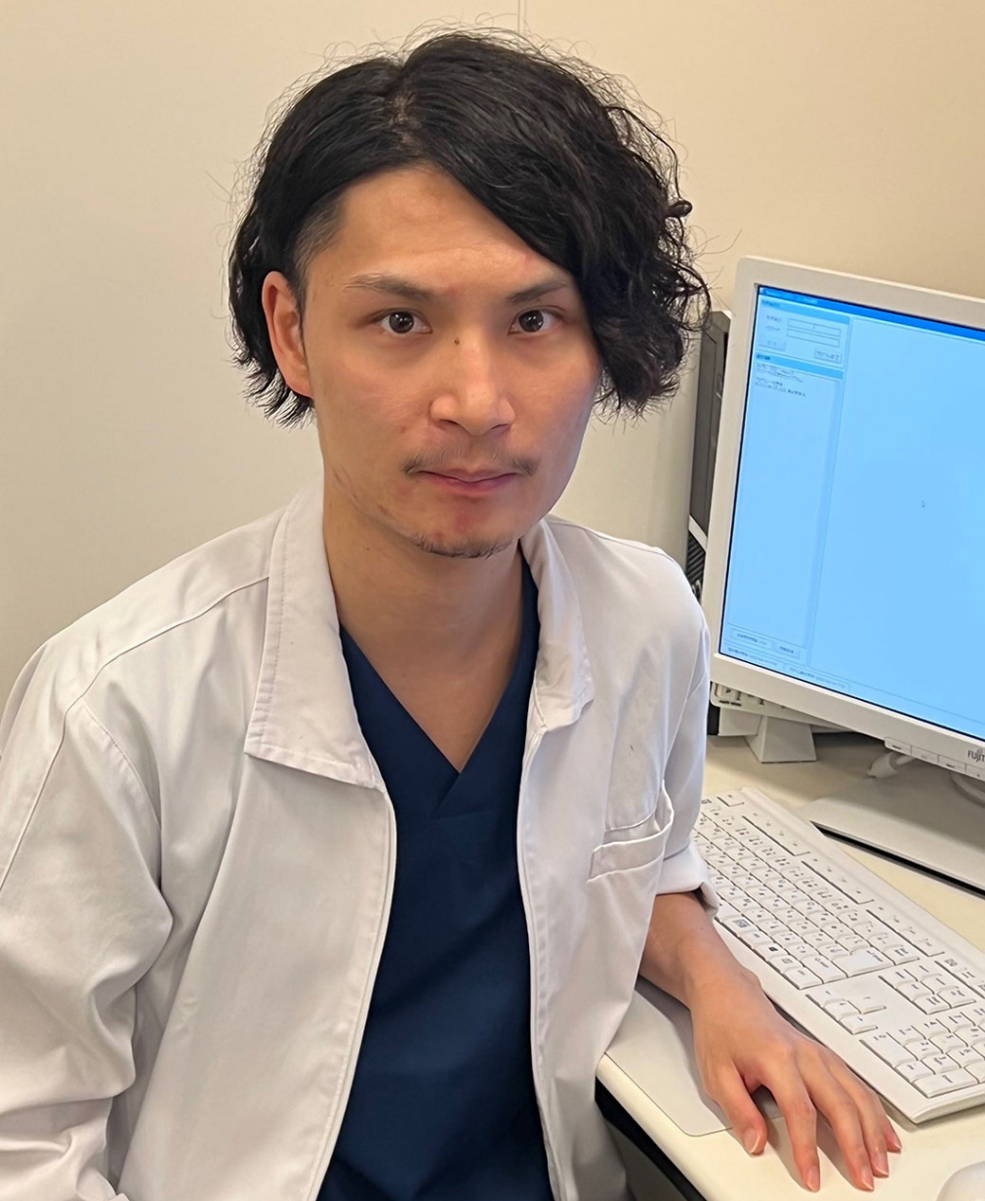


**Takashi Owaki**

**How would you explain the main findings of your paper to non-scientific family and friends?**

The prevalence of nonalcoholic fatty liver disease (NAFLD) is increasing, and its diverse and complicated etiology, ranging from obesity and dyslipidemia to abnormal hormone secretion, genetic factors and gut-liver axis, has rendered defining a standard therapeutic method rather challenging. As previously reported, autonomous nerve signals appear to be involved in the pathogenesis and progression of NAFLD through the inter-organ neural axis, but the mechanism remains unexplained, especially from a therapeutic perspective. In this study, we focused on the involvement of serotonin (5-HT), a multifunctional bioamine with important signaling roles in various physiological pathways, in NAFLD pathology via the autonomic nerve circuit. We analyzed 5-HT expression in the small intestine, its receptors (HTRs) in the liver and the expression of lipid metabolism-related genes using various NAFLD animal models with/without autonomic neural signal blockade of the liver-gut autonomic neural axis. Hepatic neural blockade retarded the progression of NAFLD by reducing 5-HT in the small intestine, hepatic HTR2A and hepatic lipogenic gene expression. Surprisingly, commercially available HTR2A antagonists blocked these therapeutic effects on NAFLD liver pathology. In addition, these effects were milder in melanocortin 4 receptor knockout (MC4R KO) mice, which have poor inhibitory control of appetite in the brain, and cerebral 5-HT and HTR2C expression did not correlate with the peripheral neural blockade. Overall, our study demonstrates that the autonomic liver-gut peripheral neural axis is involved in the etiology of NAFLD with the key players of 5-HT and HTR2A as effectors. This implies that the modulation of the axis and the use of HTR2A antagonists are potentially novel therapeutic strategies for NAFLD treatment.

**What are the potential implications of these results for your field of research?**

Based on the results of this study and our previous studies, we believe that the importance of the autonomic neural signaling pathway linking various organs, such as the liver, brain and gut, is remarkable in NAFLD pathology and as a therapeutic target. As the inter-organ pathway is conserved for the maintenance of the biological homeostasis, our study suggests that the modification/reversal of the signaling pathway is a key therapeutic target to improve the NAFLD condition, which is dramatically increasing worldwide.

“The importance of the autonomic neural signaling pathway linking various organs, such as the liver, brain and gut, is remarkable in NAFLD pathology and as a therapeutic target.”

**What are the main advantages and drawbacks of the model system you have used as it relates to the disease you are investigating?**

In this study, diet-induced NAFLD animal models were developed using wild-type mice fed with the choline-deficient defined L-amino-acid diet or with a high-fat diet (HFD) and MC4R KO mice. We used MC4R KO mice as a model to test the central melanocortin pathway in NAFLD for the following reasons: MC4R expression is limited in the brain, 5-HT and its receptor HTR2C in the brain inhibit appetite via the MC4R signaling pathway, MC4R mutations are associated with early-onset obesity and NAFLD in humans, and MC4R KO mice show the non-alcoholic steatohepatitis (NASH)-like hepatic phenotype by exhibiting an increased appetite when fed with HFD. As the effects of the autonomic neural blockade and HTR2A antagonist were milder in MC4R KO mice, and brain 5-HT and HTR2C expression were not altered with the peripheral neural blockade, further studies to elucidate the correlation between cerebral neural pathways and peripheral autonomic neural pathways are necessary.

**What has surprised you the most while conducting your research?**

We were surprised by the results that the peripheral 5-HT and HTR2A in hepatocytes are key effectors in the inter-organ communication via the hepatic-gut neural axis in the pathogenesis and progression of NAFLD, and treatment with the HTR2A antagonist clearly reproduced the results. We also confirmed the importance of inter-organ communication in liver disease via the autonomic nervous system. As we have previously reported the involvement of ghrelin as an effector in this pathway, we believe that these results shed light on the effect of gastrointestinal hormones in the pathogenesis of liver diseases.

**Figure:**
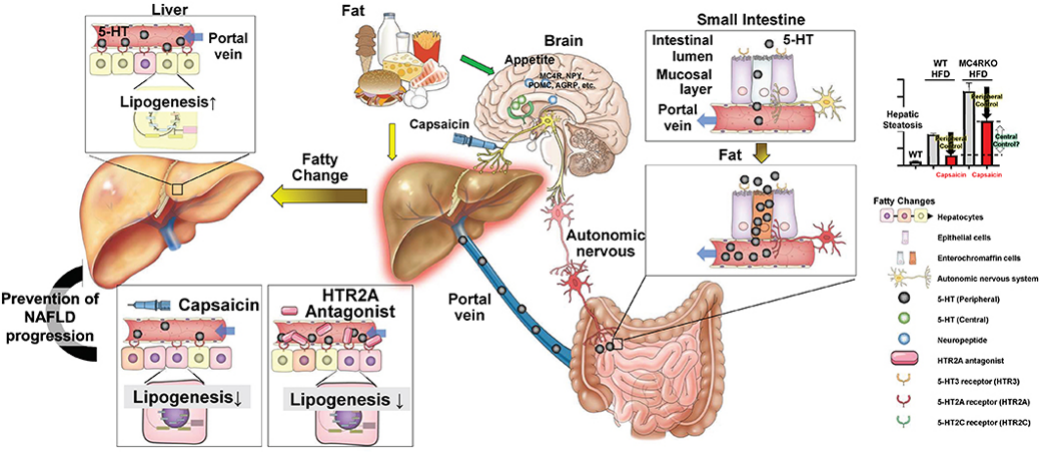
Our study demonstrates a novel role for the hepatic-gut neural axis in NAFLD progression via 5-HT and HTR2A in hepatocytes, suggesting that HTR2A antagonists are potential therapeutic agents for NAFLD.

**Describe what you think is the most significant challenge impacting your research at this time and how will this be addressed over the next 10 years?**

Based on this study, we explored the possibility of the contribution of the central 5-HT in the brain being involved in NAFLD pathology. Therefore, further experiments using mice models with impaired central neural pathways related to appetite control, involving neuropeptide Y, agouti-related protein and pro-opiomelanocortin, will further provide a novel therapeutic target for NAFLD. With the real-time monitoring of the physiological changes and neurotransmitter activation in the brain, the combination of antagonists targeting multiple pathways (e.g. HTR2A and HTR2C) will further provide information regarding the peripheral and central nervous orchestration in the NAFLD progression.

“Our research using clinically available medicines would be attractive to young physicians and contribute to increasing the number of physician-scientists in our country [Japan].”

**What changes do you think could improve the professional lives of early-career scientists?**

In Japan, it is challenging for young physicians to be interested in basic research while working in secondary care other than at a university hospital. Therefore, I believe that our research using clinically available medicines would be attractive to young physicians and contribute to increasing the number of physician-scientists in our country. In addition, by focusing on multiorgan communications, collaborative research can be conducted with researchers from various fields.

**What's next for you?**

In terms of this study, the next step is to clarify the contribution of appetite control and the relationship between the central and peripheral autonomic neural pathways in the pathogenesis and progression of NAFLD. For my career as a gastroenterologist, I want to broaden my view with these research experiences, focusing on the relationship of multiple organs in the disease's pathology and contributing to developing novel therapeutic options for various diseases.

**Would you like to acknowledge any scientists or collaborators?**

I want to thank Kenya Kamimura and Professor Shuji Terai at the Division of Gastroenterology and Hepatology at Niigata University for their continued guidance. I would also like to thank Masayoshi Ko, Itsuo Nagayama, Takuro Nagoya, Osamu Shibata, Chiyumi Oda, Shinichi Morita, Atsushi Kimura, Takeki Sato, Toru Setsu, Akira Sakamaki, Hiroteru Kamimura and Takeshi Yokoo at the Division of Gastroenterology and Hepatology at Niigata University for their cooperation with my research. I want to acknowledge Takao Tsuchida for his excellent assistance with histological analyses, as well as Nobuyoshi Fujisawa, Kanako Oda, Shuko Adachi, Toshikuni Sasaoka and all staff members at the Division of Laboratory Animal Resources at Niigata University.
